# Development of a French Paper-and-Pencil Implicit Association Test to Measure Athletes’ Implicit Doping Attitude (IAT-Dop)

**DOI:** 10.5334/irsp.651

**Published:** 2023-06-12

**Authors:** Valentine Filleul, Fabienne d’Arripe-Longueville, Eric Meinadier, Jacky Maillot, Derwin K.-C. Chan, Stéphanie Scoffier-Mériaux, Karine Corrion

**Affiliations:** 1 UniversitéCôte d’Azur, LAMHESS, FR; 2 French Federation of Cycling, FR; 3 The Education University of Hong Kong, HK

**Keywords:** implicit attitudes, doping in sport, indirect test, paper & pencil SC-IAT-P

## Abstract

Although explicit measures of doping attitude are widely used, they are susceptible to bias due to social desirability. The current computerized measures of implicit attitudes are time-consuming and based on expensive software solutions. Recently, paper-and-pencil (p&p) Implicit Association Tests (IAT) have been developed, making it possible to test several participants simultaneously, anywhere, and with no need of computerized equipment and software. The present series of studies aimed at developing a French version of a p&p IAT to measure athletes’ attitudes toward doping (Chan et al., 2017): the IAT-Dop.

Four studies, including 212 participants (*M*age = 25.49, *SD* = 5.73), followed Bardin et al. (2016) and Boateng et al. (2018) validation recommendations: (a) development of a preliminary version of the IAT-Dop based on the proposal of Chan’s tool (2017), (b) dimensionality and criterion validity tests demonstrating the structure of the p&p version, (c) test-retest reliability, and (d) first approach to construct validity.

The results showed that the IAT-Dop was able to measure implicit attitudes toward doping and was stable across time. Significant correlations between the computerized and p&p versions confirmed the construct validity. The p&p IAT-Dop showed several advantages over the computerized version (Lemm et al., 2008), including lower cost and ease of administration. By offering accurate measures and an easier, faster, and cheaper way to measure doping attitudes, this tool should contribute to the better assessment and understanding of the mechanisms related to doping, and it might be a useful new indicator in the evaluation of prevention programs.

## Introduction

The recent literature has shown that the use of illicit performance-enhancing substances remains high in sport (Lazuras et al., 2017; World Anti-Doping Agency, WADA, 2018), thus confirming the importance of sustained antidoping efforts. In the framework of socio-cognitive theories, the role of psychosocial variables related to doping in athletes (e.g., attitudes, motivation, self-regulation) has mainly been studied from the perspective of the Theory of Planned Behavior (TPB; Ajzen, 1985, 1991), the Self Determination Theory (SDT; Ryan & Deci, 2000) and the Social Cognitive Theory of Moral Thought and Action (Bandura, 1991; Bandura et al., 2001; Bandura & Locke, 2003). The TPB has emerged as one of the most influential models applied to examine doping intention and behaviors (e.g., Lucidi et al., 2008; Ntoumanis et al., 2014; Zelli et al., 2010). In their extended model of the TPB, Chan et al. (2015b) identified modal salient behavioral, normative and control beliefs, demonstrating notably that beliefs about the advantages of using banned substances positively predicted doping attitudes and intentions. Within the context of the TPB, explicit attitudes (i.e., evaluative judgements on a topic resulting from conscious processes and deliberated thoughts) were found to be the most important predictors of doping (Brand et al., 2014; Kraus, 1995).

The methods for assessing explicit attitudes toward doping are based on self-reported measures, which remain the most commonly used measures in prevention programs (e.g., the Performance Enhancement Attitude Scale, Petróczi & Aidman, 2009; the Doping Willingness in Sport Scale, Stanger et al., 2020). Although the TPB successfully contributes to predicting health behaviors (e.g., Hamilton et al., 2020), Sheeran (2005) concluded his review indicating that not more than 25 to 30% of the behavioral variance could be explained by social-cognitive variables from this line of modeling. In addition, although measures of explicit attitudes are widely used, they nevertheless present some limitations, especially when applied to a transgressive topic. Notably, as doping is illegal and perceived as socially unacceptable, it is generally acknowledged that athletes with positive attitudes toward doping are less likely to voluntarily reveal them (Brand et al., 2014; Chan et al., 2017, 2018). It is therefore understandable that athletes answering doping questions would tend to display a social desirability bias (Brand et al., 2014). Their answers depend on what they can admit (self-representation bias) and also what they are able to tell (introspective ability limitations, Blaison et al., 2006).

Such limitations might have partly contributed to the development of research on implicitness, observed in the last two decades (Corneille & Hütter, 2020). Recent dual-process theories on behaviors draw a distinction between explicit and implicit attitudinal components, such as in the Reflective Impulsive Model (Strack & Deutsch, 2004) in which authors argued that behaviors would be influenced by both reflective (explicit) and automatic cognitive (implicit) processes. Other studies on dual-process models of health behavior (e.g., Friese et al., 2011; Phipps et al., 2021) also integrate implicit mechanisms as a key determinant of behavior.

Greenwald and Banaji (1995) initially defined implicit attitudes as ‘introspectively unidentified (or inaccurately identified) traces of past experience that mediate favourable or unfavourable feeling, thought, or action toward social objects’ (p. 8). The definition of ‘implicit’ has been highly controversial within the scientific community (De Houwer, 2008; De Houwer et al., 2009; Gawronski, 2009; Nosek et al., 2007b). Corneille and Hütter warned in a recent review (2020) against a common confusion between the three conceptualizations of implicitness construct: (a) the procedural conceptualization (i.e., indirect measure), (b) the functional conceptualization (i.e., automatic responses), and (c) the mental theory (i.e., association). The initial authors recently provided a more careful definition of ‘implicit’:

Understanding 1 treats implicit and explicit as properties of psychological measures, describing measures that assess a construct indirectly (implicitly) versus directly (explicitly). Understanding 2 treats implicit and explicit as properties of mental processes or mental representations, which may be conceived as operating in automatic or unconscious fashion (implicitly) or in controlled or conscious fashion (explicitly) (Greenwald et al., 2022).

Perugini et al. (2010) reviewed the evidence that implicit measures predict behaviors. Indirect tests, based on reaction times, have been shown to measure the implicit component of attitude (Brand et al., 2014; Greenwald et al., 1998). Interest in these measures has grown over the past two decades in social psychology (see Perugini et al., 2010; Kurdi et al., 2019). Among the implicit measuring tools, the Implicit Association Test has attracted the most interest from the research community (e.g., Extrinsic Affective Simon Task (EAST), De Houwer, 2003; Go/No-Go Association Task (GNAT), Nosek & Banaji, 2001; IAT, Greenwald et al., 1998; Single Category Implicit Association Test (SC-IAT), Karpinski & Steinman, 2006). The IAT is also the most documented (Teige-Mocigemba et al., 2010), and its reliability has been demonstrated in the literature (Greenwald et al., 2009; Hofmann et al., 2005; Lotz & Hagemann, 2007; Nosek et al., 2007b). Bardin et al. (2016) offered the following simple explanation: ‘based on latency, the IAT is a categorization task of two opposite attitude objects or target concepts (e.g., insects vs. flowers) and two opposite evaluative attributes (e.g., negative vs. positive) usually used on computer’ (Bardin et al., 2016). Existing IATs have been used in several fields, such as racial prejudice, marketing, addiction behaviors, politics, and even physical activity (Bardin et al., 2016; Chevance et al., 2017; Lane et al., 2007). Among the association-based tools, the Single Category Implicit Association Test (SC-IAT) measures the strength of evaluative associations with a single attitude object (Karpinski & Steinman, 2006). Chan et al. (2018) recently used a computerized version of the brief SC-IAT (Karpinski & Steinman, 2006; Sriram & Greenwald, 2009), adapted to doping. More precisely, the authors predicted athletes’ behaviors to avoid unintentional doping. In this study, athletes with higher implicit doping scores were less likely to read the ingredients of a product, while they were more likely to report being aware of unintentional doping risk. Authors highlighted once again the need to measure both explicit and implicit attitudes regarding such topics, where athletes can report positive attitudes towards doping avoidance, while holding negative attitudes.

Although implicit measures provide important improvements in the understanding of behaviors (Perugini, 2005; Perugini et al., 2010), they should be considered with caution. First, limitations of extrapersonal association contamination in IATs (Olson & Fazio, 2004) and their relative contrasts (Swanson et al., 2001) have been highlighted in the literature. Also, implicit measures have been shown to generally provide low-to-moderate reliabilities (Greenwald & Lai, 2020) and they are usually not properly validated. In addition, IATs are traditionally run on computers (Brand et al., 2014; Petróczi et al., 2008; Schindler et al., 2015) using specialized experimental software (e.g., E-Prime; Inquisit). This type of administration presents some limitations. Indeed, it requires strict conditions to record participant reaction times. This involves experimental constraints such as dedicated rooms, computers equipped with the software, and a restricted number of participants that can be tested simultaneously, making data collection time-consuming and complicating the protocol and recruitment portability (Bardin et al., 2016; Lemm et al., 2008). In some conditions, these computerized tests are even impossible to run (e.g., playgrounds, swimming pools; Chan et al., 2017).

To overcome these limitations, paper-and-pencil versions of the IAT (p&p IAT) have been developed, based on the same logic as the computerized tests (Bardin et al., 2016; Lemm et al., 2008). These p&p versions unquestionably offer ease-of-administration prospects because, as opposed to the computerized versions, measures are obtained more quickly and easily from larger participant groups without the above-mentioned constraints. Despite these advantages, p&p versions of IAT tests are less often used, probably due to the lower accuracy compared to the computerized versions (Bardin et al., 2016). For example, the validation procedure remains flexible, is not fully codified, and sometimes lacks rigor and validity. In 2017, Chan et al. (2017) proposed a p&p IAT measuring athletes’ attitudes toward doping. This Single-Category (SC) IAT measures a single target concept: doping. SC tests are particularly suited to measuring a non-relative attitude such as doping, with simplified instructions and reduced administration time (Bardin et al., 2016).

The role of socio-cognitive variables in doping has been well documented (e.g., Chan et al., 2015a; Lucidi et al., 2008; Ntoumanis et al., 2014). However, the role of implicit processes deserves to be further explored. Currently, no validated tool can be used to assess implicit attitudes toward doping in French athletes. While the scientific community has become increasingly sensitive to the interaction between explicit and implicit processes, leading to a wider use of implicit measuring tools, their selection remains too often not supported by a solid theoretical rationale (Zenko & Ekkekakis, 2019). Worse still, some papers have based their statements on associative measures exhibiting unsatisfactory psychometric properties (Zenko & Ekkekakis, 2019). The moderate reliabilities provided can therefore come from bias in the interpretation of the results, and/or, the inadequate procedures used (Greenwald et al., 2022). An accurate and strong testing of psychometric properties of such tools is therefore crucial.

The aim of this study was thus to test and provide a preliminary validation of a French paper-and-pencil IAT as an alternative method to measure implicit attitudes toward doping. We refer to it as the ‘IAT-Dop’ for readability. Our validation was based on the proposal of Chan et al. (2017) for the topic and the design, and on the testing procedure of the paper-and-pencil Personalized Single-Category IAT test (i.e., p&p SC-IAT-P) of Bardin et al. (2016), enriched with several parts of Boateng et al.’s (2018) validation recommendations (i.e., item scale development, dimensionality, reliability, and a suggested test of construct validity). Our intention was to develop a reliable and temporally stable tool in a p&p version that would be fully congruent with the computerized IAT.

This paper was built around four studies: (a) the first study consisted in the development of a preliminary version of the IAT-Dop, (b) the second study measured the dimensionality and criterion validity of the IAT-Dop to confirm the structure of the p&p version, (c) the third study verified its test-retest reliability, and (d) the fourth study explored the relations between the p&p and computerized versions of the IAT-Dop as a first approach to construct validity.

## Study 1

The purpose of the first study was to (a) test and provide a preliminary validation of the IAT-Dop based on the SC-IAT-P (Bardin et al., 2016) and the p&p IAT proposal (Chan et al., 2017) and (b) verify its content clarity in a sample of French athletes.

### Method

#### Participants and procedure

A sample of 53 volunteer cyclists (39 males and 14 females, *M*age = 37.77, *SD* = 13.41) were recruited. They were regular cycling practitioners, and 81.1% of them were competitors (*n* = 48), including 12 professionals (22.6%). They trained about eight hours per week on average, with 45.3% training more than 10 hours per week (*n* = 24) and 5.7% training less than three hours (*n* = 3).

As the p&p IAT already has an English version, a committee approach was adopted with the most efficient composition recommended by Boateng et al. (2018). This committee was composed of nine researchers: (a) French-speaking and bilingual researchers (*n* = 4), (b) bilingual native English-speaking researchers from the United States and South Africa (*n* = 2), (c) one native French-speaking linguistics expert, and (d) the author of the original proposal of a p&p SC-IAT with doping as target (Chan et al., 2017). Two other professionals, sports physicians from the French Cycling Federation, were included in this committee for the content clarity analysis.

The first step consisted of the translation of the p&p IAT adapted to doping proposal (Chan et al., 2017). Two criteria guided the translation process: (a) conformity with the original questionnaire intentions and (b) clarity of the items in the French language (Boateng et al., 2018). The first translation into French of the p&p IAT proposal (Chan et al., 2017) was performed by the four initial researchers. Then, the French version was back-translated into English without the help of the original version by the two native English-speaking researchers. The initial researchers resolved disagreements by discussion and with the help of the linguistics expert. The author of the original proposal was consulted for any relevant questions.

Particular attention was paid to the choice of the stimuli terms because IAT quality is strongly dependent on the chosen stimuli (Fiedler et al., 2006). Decisions were made to use language that would make the items understandable to athletes and to use International French to avoid colloquialisms. Thus, the terms were first discussed with the two committee physicians, both experts on cycling. Last, a list of 14 substances was drawn up (see Tables 1 and 2) according to: (a) the initial English proposal (Chan et al., 2017), (b) the WADA classification, (c) the popularized tools of the WADA, and (d) the terms used in the French literature to talk about doping substances (e.g., Duclos, 2012; Fouillot, 2005). This list was tested with a pool/sample of 53 cyclists through an online questionnaire. The cyclists were asked to spontaneously name four words or groups of words that were the best references to doping substances. Then, they were asked to assess the relevance of the 14 terms related to doping on a 4-point Likert scale (ranging from 1 ‘completely not relevant’ to 4 ‘completely relevant’). They were finally asked to classify eight words out of the 14, from the most relevant to the least relevant (see Table 2).

**Table 1:** Words referring to doping substances according to the participants for the content clarity analysis (*n* = 53).


TERMS ‘WORDS REFERRING TO DOPING SUBSTANCES’ TERMS	OCCURRENCE (*n*)	OCCURRENCE (%)

EPO	33	31.1

Hormones	14	13.2

Transfusion	13	12.3

Anabolic [*anabolisants*]	10	9.4

Steroids [*stéroïdes*]	8	7.5

Corticoids [*corticoïdes*]	7	6.6

Amphetamines [*amphétamines*]	5	4.7

Cortisone	3	2.8

Ventoline	2	1.9

Injections	2	1.9

Cannabis	2	1.9

Speed [*excitants*]	1	0.9

Tramadol	1	0.9

Drugs [*drogue*]	1	0.9

Cocaine [*cocaine*]	1	0.9

Kenacort	1	0.9

Opiates [*opiacé*]	1	0.9

Ephedrine [*éphédrine*]	1	0.9


**Table 2:** Relevance classification for the words referring to doping substances according to the participants for the content clarity analysis (*n* = 53).


TERMS	*‘DO THESE TERMS EVOKE DOPING FOR YOU?’ (FROM 1 ‘NOT AT ALL’ TO 4 ‘TOTALLY’)* M (*SD*)	‘PLEASE RANK THESE TERMS FROM 1 TO 8 FROM THE MOST TO THE LEAST EVOCATIVE OF DOPING’ OCCURRENCE AS THE 1^ST^ ONE *n* (%)

EPO	**3.89 (0.47)**	**34 (61.2)**

Anabolic steroids	**3.83 (0.55)**	**10 (18.9)**

Growth hormone	**3.75 (0.68)**	**6 (11.3)**

Amphetamines	**3.74 (0.62)**	5 (9.4)

Corticoids	3.60 (0.72)	4 (7.5)

Masking agents	3.53 (0.85)	5 (9.4)

Stimulants	3.38 (0.71)	3 (5.7)

Morphine	3.15 (0.93)	1 (1.9)

Beta-blockers	3.09 (0.97)	**7 (13.2)**

Cocaine	3.04 (1.04)	3 (5.7)

Narcotics	2.74 (1.21)	4 (7.5)

Cannabis	2.66 (1.06)	1 (1.9)

Diuretics	2.53 (1.14)	2 (3.8)

Marijuana	2.51 (1.10)	1 (1.9)


*Notes*. Relevance scores on a 4-point Likert scale.

### Results

The committee reached consensus regarding the following words: (a) for the category ‘I like’: *bonheur* (happy), *plaisir* (pleasure), *amour* (love), and *liberté* (freedom) and (b) for the category ‘I dislike’: *mal* (evil), *puanteur* (stink), *saleté* (filth) and *accident* (accident).

**Figure 1:**
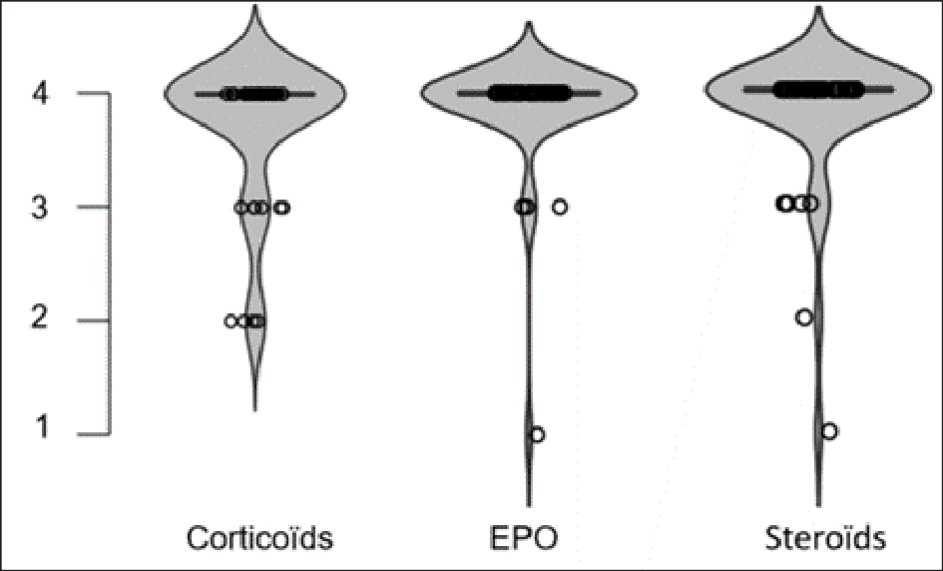
Boxplots describing relevance scores on a 4-point Likert scale for the stimuli words: Corticoïds, EPO and Steroïds, (*n* = 53). *Notes*. For the 4-point Likert-scale: 4 = ‘Totally,’ 3 = ‘A little,’ 2 = ‘Not really,’ 1 = ‘Not at all.’

Regarding the choice of target category, the proposed stimuli (see Tables 1 and 2, Figure 1) obtained a mean relevance score of 3.25 (*SD* = 0.86). The term *EPO* [EPO] was retained, as it appeared to be the most significant. The term *stéroïdes anabolisants* [anabolic steroids] was also selected but after discussion it was decided to keep only *stéroïdes* [steroids] and delete *anabolisants* [anabolic] both to be congruent with the original proposal (Chan et al., 2017) and to ensure size uniformization of the four stimuli terms. Also, we decided to keep *corticoïdes* [corticoids], which is easier to read in French than *amphétamines* [amphetamines].

Ultimately, only one word from the original proposal was kept for the target category (Chan et al., 2017): ‘steroids,’ and the three other words were selected following the content clarity and relevance analysis. We added as the fourth word, *transfusion* [transfusion] because it was the word the third most frequently mentioned in the free text question, and we wanted to reflect better the WADA prohibited list which includes both substances (e.g., anabolic agents, peptide hormones) and methods (e.g., manipulation of blood and blood components, chemical and physical manipulation. The committee unanimously approved the choice of this word (i.e., transfusion).

The preliminary French translation was satisfactory (Boateng et al., 2018) because the English back-translation from our committee of experts was very close to the original proposal of the IAT and adapted to the population (Chan et al., 2017).

### Discussion

This first study enabled us to provide a preliminary version of the p&p IAT-Dop based on the existing p&p SC-IAT-P (Bardin et al., 2016) and Chan et al. (2017) proposal. The clarity and relevance reliability of the tool were demonstrated. At this step, the IAT-Dop was transculturally translated into French. This tool was adapted for a population of cyclists due to the high prevalence of doping and the specific history of doping in this sport. However, the next steps of the validation procedure were conducted with both high-level cyclists and university students practicing a variety of sports to ensure the broader usability of the tool.

## Study 2

Following both the recommendations of Boateng et al. (2018) and the SC-IAT-P testing procedure (Bardin et al., 2016), the aim of this second study was to measure the dimensionality and criterion validity of the IAT-Dop. This step included the examination of the IAT-Dop structure and assessed the criterion validity of the IAT-Dop by highlighting relationships between this tool and explicit variables.

### Method

#### Participants

One hundred and fifty-nine French-speakers, either students enrolled in a university school of sports science or high-level cyclists, took part in this study (*M*age = 21.39, *SD* = 3.17). This sample included 119 male (*M*age = 21.48, *SD* = 2.46) and 40 female (*M*age = 21.08, *SD* = 4.71) volunteers. They were recruited from a university in the south of France during classes and from a high-level cycling center during a training camp. They averaged 8.2 hours of training per week (*SD* = 4.6). As the participants came from a university sports science school and a cycling training camp, various sports were practiced and many of the participants were practicing several sports simultaneously (e.g., collective sports like football or handball, as well as individual sports like cycling, bodybuilding or running). A sensitivity power analysis with GPower 3.1 software, assuming an α of 0.05 and a power of 0.80, indicated that the minimum effect size we had power to detect was a moderate-to-large effect of 0.60. Similar studies on p&p implicit measurement tools have been conducted with similar sample size (Bardin et al., 2016; Lemm et al., 2008).

#### Procedure

The tests were conducted by two researchers (i.e., the main researcher and a trainee). Participants were alone with the researcher in a small room with no sources of distraction and in total silence. A booklet was given to them. It included instructions, the items for the measure of implicit doping attitudes followed by a self-report doping behavior questionnaire, and sociodemographic data. We put into place both the minor assent forms and the major consent forms. Participants were aware that the study and the whole questionnaire, concerned the sport context and that they were enrolled because they were athletes.

#### Measures

##### The paper-and-pencil IAT-Dop

**Figure 2:**
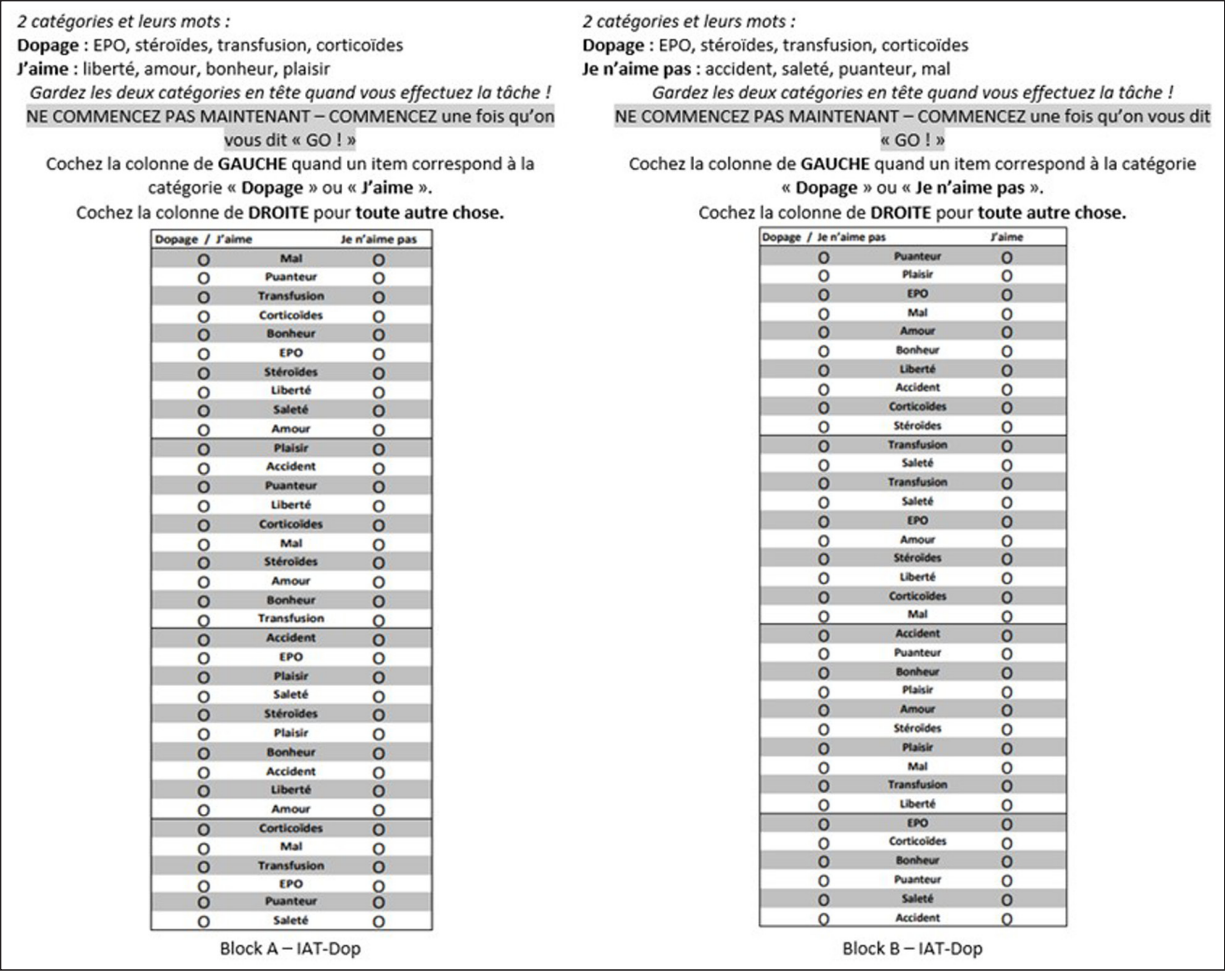
Block A (left) and Block B (right) of paper-and-pencil IAT-Dop for implicit doping attitude in the French language.

The instrument was based on the SC-IAT-P (Bardin et al., 2016) and the p&p IAT proposal (Chan et al., 2017), which show randomly placed stimuli words. To help with reading, the stimuli words appeared within a box against a gray and white background alternatively (see Figure 2) according to the recommendations of Nosek et al. (2007a). The instrument was composed of two blocks (i.e., block A ‘Doping + I like’ and block B ‘Doping + I don’t like’) which both contained a training phase and a test phase). Following the recommendations of Greenwald et al. (2022) and the p&p SC-IAT-P testing procedure (Bardin et al., 2016), the blocks A and B were counterbalanced across subjects.

Following the SC-IAT-P testing procedure (Bardin et al., 2016), participants had to categorize the stimuli words of the central column by checking the circle of the appropriate column as fast as they could in 20 seconds and with as few mistakes as possible. They received all the instructions before starting, they could take all the time they needed between each session (i.e., training phase, test phase), to ask their questions or to get ready and focus again. The time to execute the task was the same for the two phases (i.e., 20 seconds to categorize as many words as possible). In general, participants needed five minutes to understand the instructions and to complete the training phase.

##### Self-reported doping behaviors

Self-reported doping behaviors were assessed with a list of the most frequently used substances in sport, both legal and prohibited. This list was created by a committee composed of the four initial researchers and the two physicians who were cycling experts, previously cited in Study 1, and was based on: (a) the substance list from the WADA Code, (b) the French National Agency for Food, Environmental and Occupational Health & Safety and (c) the National Syndicate of Supplements (Synadiet). Participants were asked yes-no questions on whether they had used any of the substances in the past three months, either legal (i.e., effort drinks, vitamins and minerals, energizing drinks, protein powder, homeopathy, phytotherapy and herbal medicines, creatine, and non-prescription drugs) or prohibited (i.e., diuretics, stimulants, glucocorticoids, cannabinoids, and anabolic agents). Researchers also reduced order effects by systematically varying the order in these substance use questionnaires so that each condition was presented equally often in each ordinal position (Morling, 2015). Half of the participants responded to the questionnaire on substance use before the whole IAT procedure, and the other half responded at the end, just before the sociodemographic questionnaire. The order did not impact the results (*r* = –0.00, *p* = 0.96; *t*(142) = 0.05, *p* = 0.96, *d* = 0.01).

Two groups of participants were constituted based on their self-reported doping behavior. According to the WADA Code (2021), those participants admitting to the use of at least one of the prohibited substances within the last three months, were considered to belong to the ‘dopers’ group, and all others were classified as ‘non-dopers.’

##### Sociodemographic data

The sociodemographic data sought from the participants concerned their personal situation (i.e., gender, age, nationality, relationship), and sports characteristics (i.e., sport level, years of experience, weekly training volume).

### Data analysis

#### Descriptive analysis

The strength of any association between concepts was measured by the standardized mean difference score of the ‘hypothesis-inconsistent’ pairings (i.e., ‘Doping’ + ‘I like’) and ‘hypothesis-consistent’ pairings (i.e., ‘Doping’ + ‘I don’t like’), called the *d*-score (Greenwald et al., 2003). For the p&p version, several calculations are possible to obtain the implicit attitude score, known as the *d*-score. However, the researchers agreed that the procedure recommended by Lemm et al. (2008) would give the most reliable results. The *d*-score consists of the product of the square root of the difference, which considers the number of stimuli correctly checked for the first block, called A (‘I like + Doping’ combination) and the number of stimuli correctly checked for the second block, called B (‘I don’t like + Doping’ combination). The score is obtained by this calculation expression: if A > B, D = A/B∗√ |A–B|; if B > A, D = B/A∗ (–1)∗√ |A–B|. A positive *d*-score supports a stronger association between ‘I like – Doping’ than ‘I don’t like – Doping’ with the magnitude of the score indicating the strength of the implicit attitude. When participants had an error rate of more than 20% (Karpinski & Steinman, 2006), their data were excluded from the analyses.

#### Statistical analysis

Gender, age, sports characteristics (i.e., sport level, years of experience, weekly training volume), self-reported doping behaviors, and IAT block order were controlled for in this analysis through linear regressions. After assessing the normality of the distribution, a *t*-test for independent samples was used to compare the implicit attitude score between two groups (i.e., the ‘non-dopers’ and ‘dopers’ groups). We tested the alternative hypothesis that the implicit attitudes (*d*-scores) toward doping of ‘non-dopers’ would be weaker (i.e., lower *d*-scores) than those of ‘dopers’ (McKenzie, 1998). Scores of each group were then compared to zero with single sample *t*-tests. Statistical analyses were performed with SPSS (IBM Corporation, version 25) software.

### Results

Fifteen participants (9.43%) presented error rates > 20% and were excluded from the analysis. Analyses were thus run on 144 participants (*M*age = 21.2; *SD* = 2.80; 110 males and 34 females). Sociodemographic data are available in Table 3.

**Table 3:** Sociodemographic data for Study 2 and Study 4.


	Study 2			STUDY 4
	
	ALL PARTICIPANTS *n* = 144	“NON-DOPERS” GROUP *n* = 115	“DOPERS” GROUP *n* = 29	ALL PARTICIPANTS *n* = 136
	
**Age:** mean (*SD*)	21.22 (2.80)	21.28 (3.09)	21.00 (1.13)	21.22 (2.86)

**Sex**					

– Male: *n* (%)	110 (76.39)	81 (70.43)	29 (100)	105 (77.21)

– Female: *n* (%)	34 (23.61)	34 (29.57)	0	31 (22.79)

**Personal situation**					

– Single: *n* (%)	103 (71.53)	83 (72.17)	20 (68.97)	98 (72.06)

– In a relationship: *n* (%)	39 (27.08)	31 (27.00)	8 (27.59)	36 (26.47)

– Married: *n* (%)	2 (1.39)	1 (0.83)	1 (3.44)	2

– Divorced, widowed: *n* (%)	0	0	0	0

**Country of residence**					

– France: *n* (%)	143 (99.31)	114 (99.13)	29 (100)	136 (100)

– Abroad: *n* (%)	1 (0.69)	1 (0.87)	0	0

**Sport experience years**					

– In general: mean (*SD*)	14.60 (4.02)	14.77 (4.15)	13.97 (3.48)	14.05 (4.10)

– In the current sport: mean (*SD)*	9.98 (5.35)	10.09 (5.46)	9.55 (4.97)	9.86 (5.30)

– Less experience in current sport (≤10 years): *n* (%)	75 (52.08)	61 (53.04)	14 (48.28)	73 (53.67)

– Long experience in current sport (>10 years): *n* (%)	65 (45.14)	50 (43.48)	15 (51.72)	59 (43.38)

**Sport level**					

– National: *n* (%)	84 (58.33)	62 (53.91)	22 (75.86)	78 (57.35)

– International: *n* (%)	57 (39.58)	50 (43.48)	7 (24.14)	55 (40.44)

**Weekly training volume in hours:** mean (*SD*)	8.34 (4.69)	8.68 (4.99)	6.98 (3.00)	8.36 (4.67)

– Light training volume (≤10 hours/week): *n* (%)	106 (73.61)	80 (69.57)	26 (89.67)	100 (73.53)

– Heavy training volume (>10 hours/week): *n* (%)	35 (24.31)	32 (27.83)	3 (10.34)	33 (24.26)


Of the control variables on the *d*-scores for the total sample, the age (*β* = 0.135; *p* = 0.107) and the sports characteristics (i.e., sport level, *β* = 0.043; *p* = 0.615; years of experience, *β* = 0.082; *p* = 0.337; weekly training volume, *β* = –0.023; *p* = 0.784) were not significant. The gender effect on the *d*-scores was close to significance (*β* = –0.160; *p* = 0.055), with females tending to have lower *d*-scores (*n* = 34, *M* = –1.19, *SD* = 2.43) than males (*n* = 110, *M* = –0.34, *SD* = 2.18). This observation led us to rerun the analysis without females (*n* = 34). An effect of block order on the *d*-scores was observed (*β* = 0.199; *p* = 0.017), indicating that participants who began with block A tended to have higher *d*-scores (*M* = –0.05, *SD* = 2.28) than those who began with block B (*M* = –0.95, *SD* = 2.18). Finally, the effect of the self-reported behaviors on the *d*-scores was also close to significance (*β* = 0.150, *p* = 0.072), which encouraged us to consider two groups (i.e., a ‘dopers’ group and a ‘non-dopers’ group). Twenty-nine participants (20.14%) were classified in the ‘dopers’ group. All were male. The other group (i.e., ‘non-dopers’) included 115 participants (79.86%), male and female, and was composed of the participants who did not state that they had used prohibited substances. The ‘dopers’ group (*n* = 29) ticked 27.48 (*SD* = 5.10) and 26.93 (*SD* = 5.04) out of 36 items (i.e., 76.4% and 74.7%) respectively for the blocks A ‘Doping + I like’ and B ‘Doping + I don’t like.’ The ‘non-dopers’ group (*n* = 115) ticked 27.72 (*SD* = 5.14) and 29.11 (*SD* = 4.72) out of 36 items (i.e., 76.9% and 80.8%) respectively for the blocks A and B.

**Figure 3:**
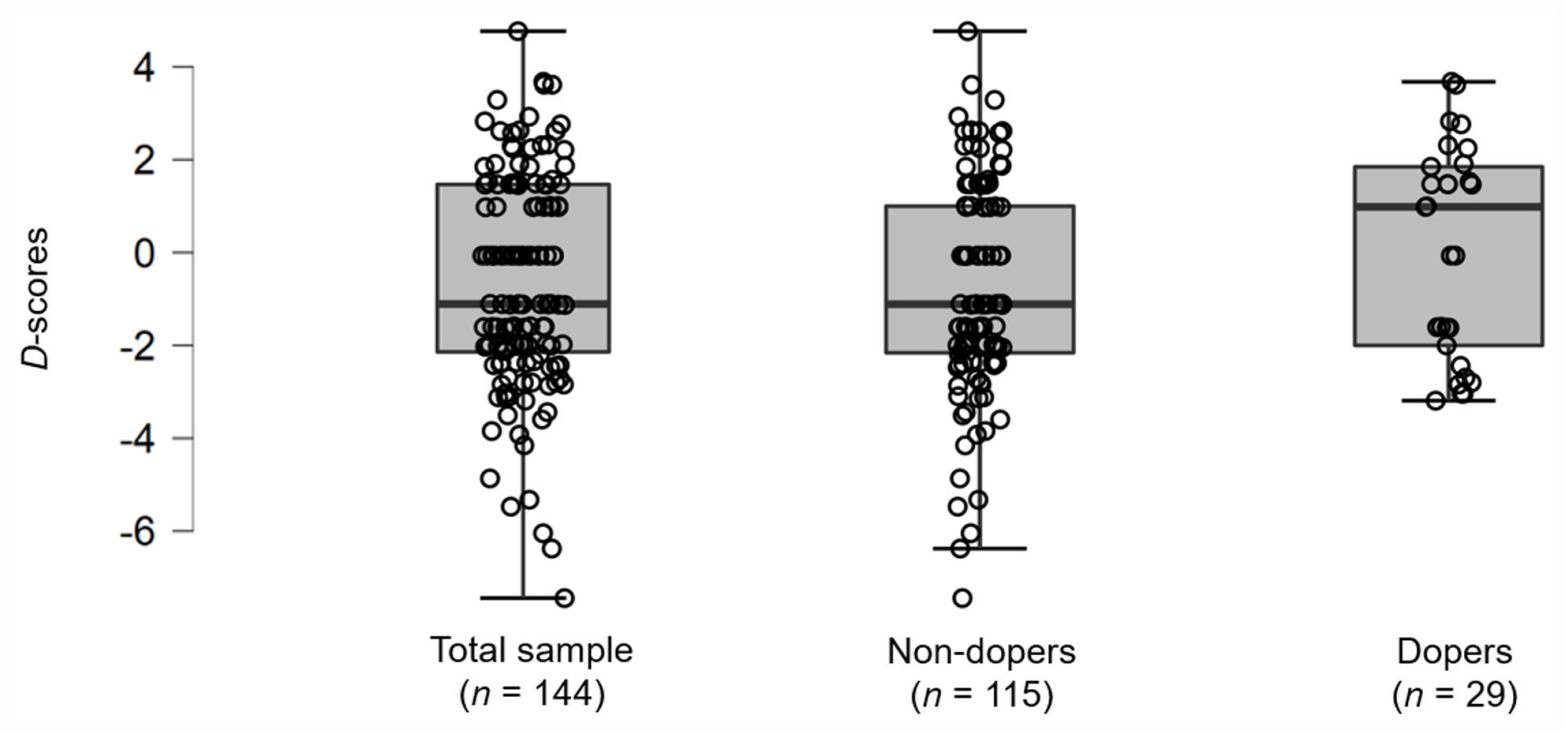
Boxplots describing the p&p IAT-Dop *d*-score distribution among groups (total sample, ‘non-dopers’ group, ‘dopers’ group).

As shown in Figure 3, the mean implicit attitude toward doping was negative for the ‘non-dopers’ group (i.e., *d*-scores: *M* = –0.72, *SD* = 2.25), as a negative *d*-score reflects a negative attitude toward doping (i.e., doping is considered undesirable). This result was congruent with the condition of the ‘non-dopers’ group. For the ‘dopers’ group, the mean implicit attitudes toward doping were positive (i.e., *d*-scores: *M* = 0.13, *SD* = 2.25), which reflected a positive attitude toward doping (i.e., doping is considered desirable). This was congruent with the condition of the ‘dopers’ group.

Using the Shapiro-Wilk test, the normality of the distribution of the scores of the ‘dopers’ group (*n* = 29) was not confirmed: W = 0.90, *p* = 0.01. We therefore used a non-parametric test (i.e., the Mann-Whitney test) for independent samples. This test confirmed the tendency previously found that the ‘dopers’ group had stronger positive implicit attitudes toward doping than ‘non-dopers’: U = 1365.50, *p* = 0.07, *rbc* = –0.18. To emphasize the previous result, scores by group were compared to 0 with single sample *t*-tests. According to the normality check, we applied a one sample *t*-test for the ‘non-dopers’ group and a Wilcoxon’s Rank test for the ‘dopers’ group. Comparing scores by group to zero, a significant difference was observed for the ‘non-dopers’ group (*t*(114) = –3.41, *p* < 0.01, *d* = –0.32) while no significant difference from 0 was observed for the ‘dopers’ group (V_w_ = 189.00, *p* = 1.00, *rbc* = 0.00).

**Figure 4:**
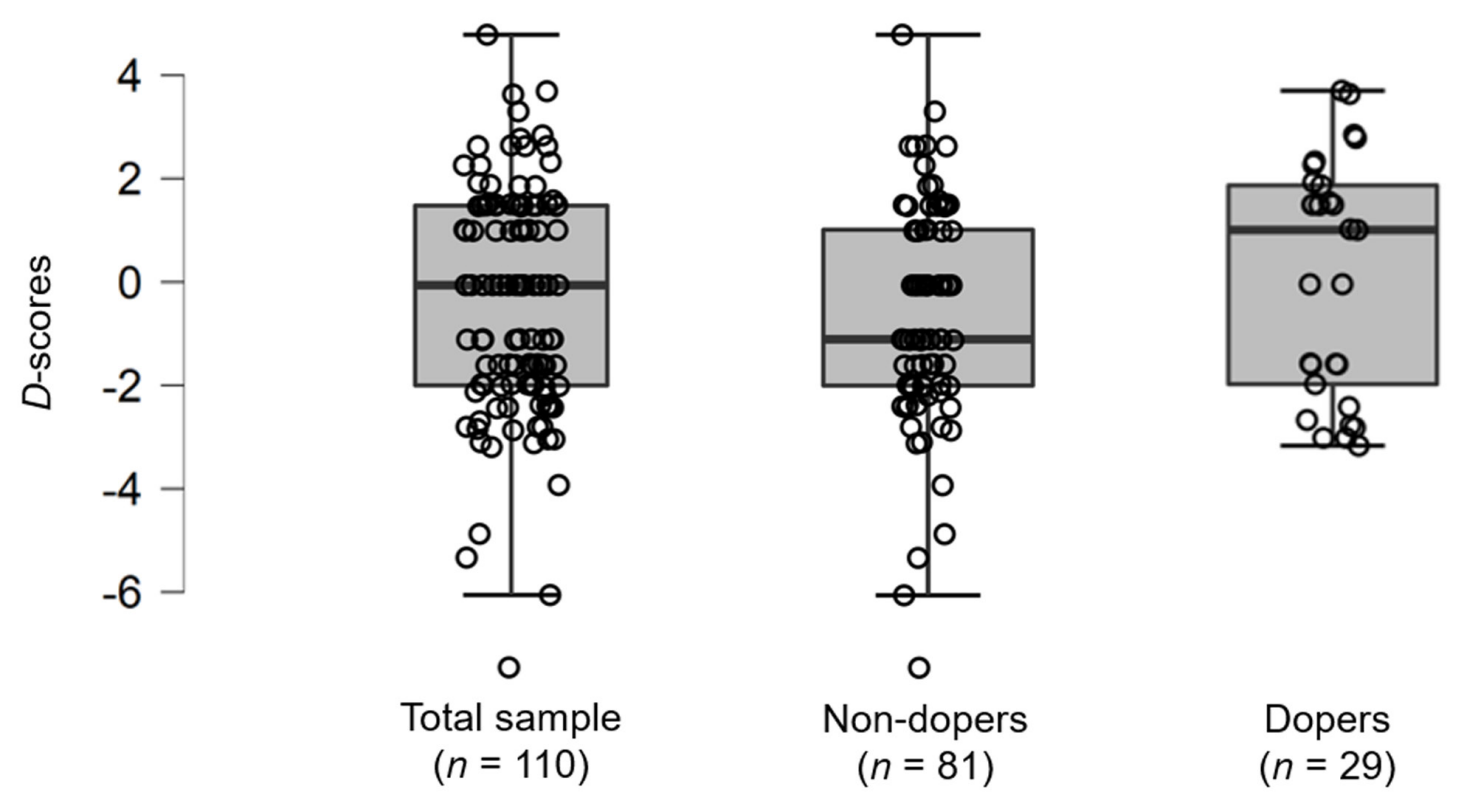
Boxplots describing the p&p IAT-Dop *d*-score distribution among groups for the male population (total sample, ‘non-dopers’ group and ‘dopers’ group).

As the group ‘dopers’ was exclusively male, and because we noticed that the gender had an effect close to significance on the *d*-scores of the total sample, we reran the analysis excluding females (*n* = 34). Figure 4 describes the *d*-scores distribution among groups for the male population (*n* = 110). Similar patterns of results were found: U = 1011.50, *p* = 0.14, *rbc* = –0.14.

Regarding the use of non-prohibited substances (e.g., vitamins, proteins), in the entire pool of participants, only two out of 144 did not use any. Participants declared having used 3.20 (*SD* = 1.87) substances in average. Among the non-prohibited substances presented in the questionnaire, the most used were non-prescription medications (*n* = 118, 81.94%), followed by vitamins and minerals (*n* = 92, 63.89%) and energizing drinks (e.g., RedBull, Monster, *n* = 79, 54.86%). There was no significant correlation between the number of non-prohibited substances used and the IAT-Dop scores (*r* < –0.01, *p* = 0.95).

### Discussion

This second study assessed the dimensionality and criterion validity of the IAT-Dop, showing its ability to measure athletes’ positive and negative implicit attitudes toward doping in sport. The IAT-Dop provided a way to measure both positive and negative attitudes toward doping in sport (Brand et al., 2014; Chan et al., 2017; Petróczi et al., 2008).

The error rate (i.e., 9.43%) was relatively high compared to similar studies (e.g., Bardin et al., 2016; Lemm et al., 2008). This phenomenon could be explained by the nature of the subject (i.e., doping): not familiar and especially sensitive, and which therefore made it particularly difficult for the participants. Furthermore, we noticed an order effect with higher *d*-scores for participants starting with block A (i.e., Doping + I like) than for participants starting with block B. This effect of order has frequently been reported in research on IAT, but it is generally weak (e.g., Clément-Guillotin et al., 2012; Greenwald et al., 2003; Nosek, et al., 2005). For a sensitive topic such as doping, it is understandable that the block A appeared as ‘more difficult’ than the block B. The participants starting with the ‘easier block’ (i.e., block B) benefited from more experience and time to get familiar with the words and the task before executing the ‘harder block’ (i.e., block A), and therefore, achieved better the second block: block A, resulting to higher *d*-scores.

Also, this study showed an association between IAT-Dop scores (i.e., *d*-scores) and doping behavior. Regarding descriptive statistics, we observed that the mean *d*-scores were higher in the ‘dopers’ group than in the ‘non-dopers’ group. However, the high standard deviations confirmed the importance of statistically testing the association between IAT-Dop scores and doping behavior. This association appeared as only a tendency, potentially due to the small sample of the ‘dopers’ group. Also, comparing scores by group to zero, we highlighted a difference of *d*-scores only for the ‘non-dopers’ group (i.e., and not in the ‘dopers’ group). In the ‘dopers’ group, the attitudes toward doping seemed to be neutral or ambivalent. The repeated analysis excluding females did not provide better results, probably due to the reduction of the sample size. Regarding descriptive statistics, we observed that the mean *d*-scores were higher in males than in females, consistent with the literature related to doping risk factors (see Nicholls et al., 2017, for a review).

This step in the validation process was nonetheless a strength of our paper, notably of our methodology, as other studies of IAT validation have not proceeded to dimensionality and criterion validity testing due to the lack of guidelines.

## Study 3

Following Boateng et al.’s (2018) validation recommendations, the aim of this study was to assess the test-retest reliability of the IAT-Dop. Only a few studies have assessed the temporal stability of the IAT, and no study to our knowledge has tested the test-retest reliability of the p&p IAT (e.g., Chevance et al., 2017; Lane et al., 2007).

### Method

#### Participants and procedure

A total of 87 sports science students (75 males and 12 females) recruited from a university in the south of France volunteered to participate in this study. Their average age was 21.39 (*SD* = 1.86) years. They practiced various sports, both collective (e.g., football, basketball) and individual (e.g., cycling, dance) and averaged 7.8 hours of training per week (*SD* = 5.00). Most of them (*n* = 63, 72.41%) were competitors at national level or below, and the others were international competitors (n = 24, 27.59%).

Participants were asked to complete the IAT-Dop twice in the same conditions (i.e., participants were alone with the researcher in a small room with no sources of distraction and in total silence), with an average time interval of two weeks (Marx et al., 2003; Vallerand, 1989). Each participant had a unique self-generated code to ensure anonymity as well as the correct pairing of the data from the two measurements.

#### Measures

Implicit attitudes toward doping were measured with the IAT-Dop. As for Study 2, the obtained scores were called *d*-scores and a positive *d*-score indicated a stronger association with ‘I like – Doping’ than ‘I don’t like – Doping.’

### Data analysis

After the normality of the data was checked, we used Pearson correlation coefficients to estimate the strength of the relationship between Time 1 and Time 2 (Lane et al., 2007). We followed the recommendations of an interval of two to three weeks (Marx et al., 2003) between the two times of measurement.

### Results

For the second measurement, nine participants were excluded from the analysis (10.34%) because they had error rates greater than 20%, and one was excluded because of a methodological problem (completion of different task order between T1 and T2). Therefore, the analyses were run on 77 participants (*M*age = 21.38; *SD* = 1.84; 67 males and 10 females). At Time 1, the participants ticked 27.44 (*SD* = 5.39) and 28.57 (*SD* = 5.17) out of 36 items (i.e., 76.1% and 79.4%) respectively for the blocks A ‘Doping + I like’ and B ‘Doping + I don’t like.’ At Time 2, the participants ticked 29.60 (*SD* = 5.00) and 29.71 (*SD* = 4.42) out of 36 items (i.e., 82.2% and 82.5%) respectively for the blocks A ‘Doping + I like’ and B ‘Doping + I don’t like.’

**Figure 5:**
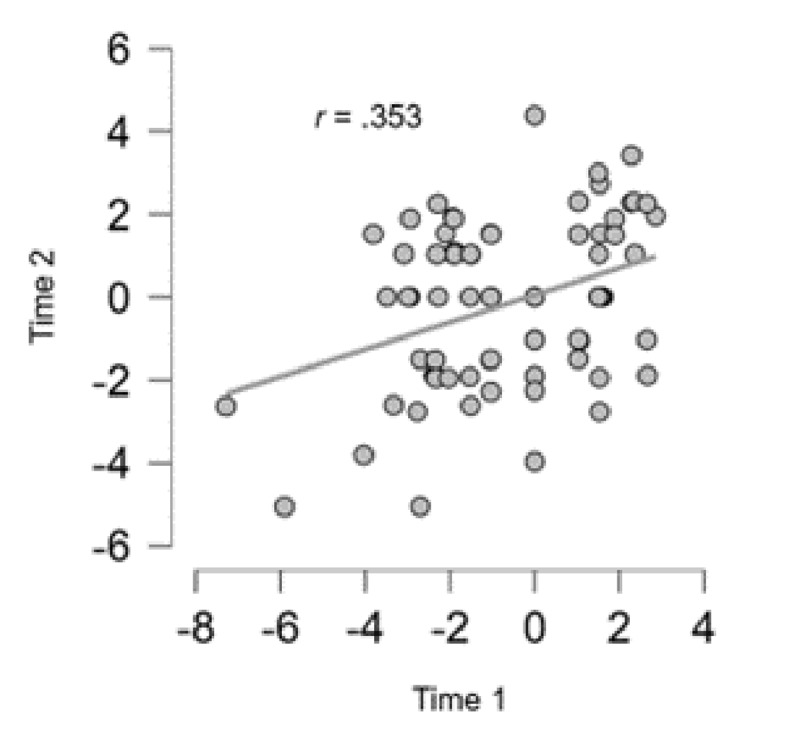
Scatter plot showing the relationship between the two measurements (Time 1 and Time 2) of *d*-score for the p&p IAT-Dop for individuals.

In this study, we analyzed the stability of the IAT-Dop over a time interval of 17.46 days (*SD* = 4.02). At Time 1, the mean *d*-score was –0.64 (*SD* = 2.13), and at Time 2, the mean *d*-score was –0.09 (*SD* = 1.90). The Pearson correlation coefficients showed significant test-retest reliability (Figure 5), *r* = 0.35, *p* < 0.01, with a statistical power of 94%, which is in line with recent recommendations in the field of IAT (Greenwald et al., 2022).

### Discussion

This study showed comparable patterns of attitudes in the test-retest. These results thus demonstrated a significant test-retest reliability, according to the recent meta-analysis on Test-Retest reliabilities of IAT (Greenwald & Lai, 2020).

In this study again the error rate (i.e., 10.34%) was relatively high, which supports the idea that the topic of doping was particularly hard for participants to approach and led to numerous mistakes. Applying Karpinski and Steinman’s (2006) exclusion criteria (i.e., excluding participants with error rates >20%) offered a strong protection from bias of misunderstandings.

This third step in the validation process was a strength of our study. To our knowledge, no study has tested the reliability of a p&p SC-IAT or p&p SC-IAT-P.

## Study 4

This study was a first approach to testing the construct validity of the IAT-Dop. This step in the validation process consisted of an examination of the link between the two versions of the tool: the p&p and the computerized IAT-Dop, following both the validation recommendations of Boateng et al. (2018) and the SC-IAT-P testing procedure (Bardin et al., 2016).

### Method

#### Participants and procedure

This sample of participants was the same as for Study 2, comprising 159 university sports science students and high-level cyclists, all volunteers, including 119 males (*M*age = 21.48, *SD* = 2.46) and 40 females (*M*age = 21.08, *SD* = 4.71). A sensitivity power analysis with GPower 3.1 software, assuming an *α* of 0.05 and a power of 0.80, indicated that the minimum effect size we had power to detect was a moderate-to-large effect of 0.60. For comparison, the two versions of the SC-IAT-P (i.e., computer and paper), Bardin et al. (2016) included 44 participants.

The protocol was the same as that employed in Study 2. Participants had to complete both computerized and p&p versions of the IAT in a counterbalanced random order which did not impact the results (*r* = 0.13, *p* = 0.13; *t*(142) = –1.53, *p* = 0.13, *d* = –0.26). Sessions lasted 10 minutes on average for the entire protocol. They received all the instructions before starting and had as much time as they needed to get ready.

#### Measures

Implicit attitudes toward doping were measured two ways: (a) with the p&p IAT-Dop as presented above (see Figure 2) and (b) with the computerized version of the IAT-Dop. The computerized IAT-Dop was based on the SC-IAT-P (Karpinski & Steinman, 2006; Olson & Fazio, 2004), enriched with the one employed by Bardin et al. (2014). Data were collected using the Milliseconds Inquisit lab 6 software. The attribute categories were *J’aime* (‘I like’) and *Je n’aime pas* (‘I dislike’). The stimuli and the items were those selected in Study 1 (i.e., EPO, transfusion, steroids, corticoids, happy, pleasure, love, freedom, evil, stink, filth, accident). Categories were counterbalanced on the computer (*r* = 0.09, *p* = 0.30), with the order of blocks respecting the order of the p&p version for each participant (e.g., if the participant started with block A in the p&p version, this participant also started with block A in the computerized version). Participants had to classify the stimuli as fast as possible, using the ‘e’ and ‘i’ keys, which are in the same row of an azerty keyboard. The implicit scores for the computerized version were calculated in accordance with the algorithm (*d*-score) proposed by Karpinski and Steinman (2006). A positive *d*-score indicated a positive attitude toward doping. Following Karpinski and Steinman’s (2006) procedure, non-responses and those given in less than 350 *ms* were eliminated. The response times for errors were replaced by the average time for the block, and a penalty of 400 *ms* was added. An error rate over 20% was considered an exclusion criterion, as recommended by Karpinski and Steinman (2006). Slow latencies (>1500 *ms*) were considered as non-responses and eliminated (Karpinski & Steinman, 2006).

### Data analysis

Gender, age, sports characteristics (i.e., sport level, years of experience, weekly training volume), and IAT block order were controlled for in linear regressions regarding the *d*-scores of each version (i.e., computerized and p&p). An initial assessment of the concurrent validity was made by examining Pearson correlations between the *d*-scores of the two versions of the tool: the p&p and the computerized IAT-Dop.

### Results

Twenty-three participants (14.47%) were excluded from the analyses. Eight of them (5.03%) presented error rates >20% for the computerized version, 14 (8.81%) presented error rates >20% for the p&p version, and one (0.63%) presented an error rate >20% for both computerized and p&p versions.

The analyses were thus run on 136 participants (*M*age = 21.2; *SD* = 2.9; 105 males and 31 females). Further socio-demographic data are available in Table 3 (Study 2). Examining the effect of the control variables on the *d*-scores for the total sample, age was not significant either on the computer (*β* = –0.026; *p* = 0.761) or on the p&p (*β* = 0.145; *p* = 0.091) versions of the test. Regarding the sports characteristics, only the sport level impacted the computer *d*-scores (*β* = –0.252; *p* = 0.003), with international athletes tending to present lower *d*-scores (*n* = 55; *M* = –0.40, *SD* = 0.34) than national athletes (*n* = 78; *M* = –0.24, *SD* = 0.30). This result could be explained by the fact that international athletes were more familiar with doping terms. None of the other variables related to the sports characteristics impacted the computer *d*-scores (i.e., years of experience: *β* = –0.067; *p* = 0.448; weekly training: *β* = –0.091; *p* = 0.297) or p&p *d*-scores (i.e., years of experience: *β* = –0.075; *p* = 0.384 and weekly training: *β* = –0.019; *p* = 0.829). The gender effect on the *d*-scores was significant for both the computer (*β* = –0.273; *p* = 0.001) and the p&p versions (*β* = –0.187; *p* = 0.029), with females (n = 31) tending to have lower *d*-scores with both the computer (*M* = –0.46, *SD* = 0.30) and the p&p versions (*M* = –1.27, *SD* = 2.44) than males (*n* = 105, respectively *M* = –0.25, *SD* = 0.32; *M* = –0.26, *SD* = 2.17). An effect of block order on the *d*-scores was observed only for the p&p version (*β* = 0.188; *p* = 0.028), which was congruent with the observations of Study 2. This result could be explained by the fact that with p&p version, they do not benefit from direct feedback as is the case with computerized versions.

**Figure 6:**
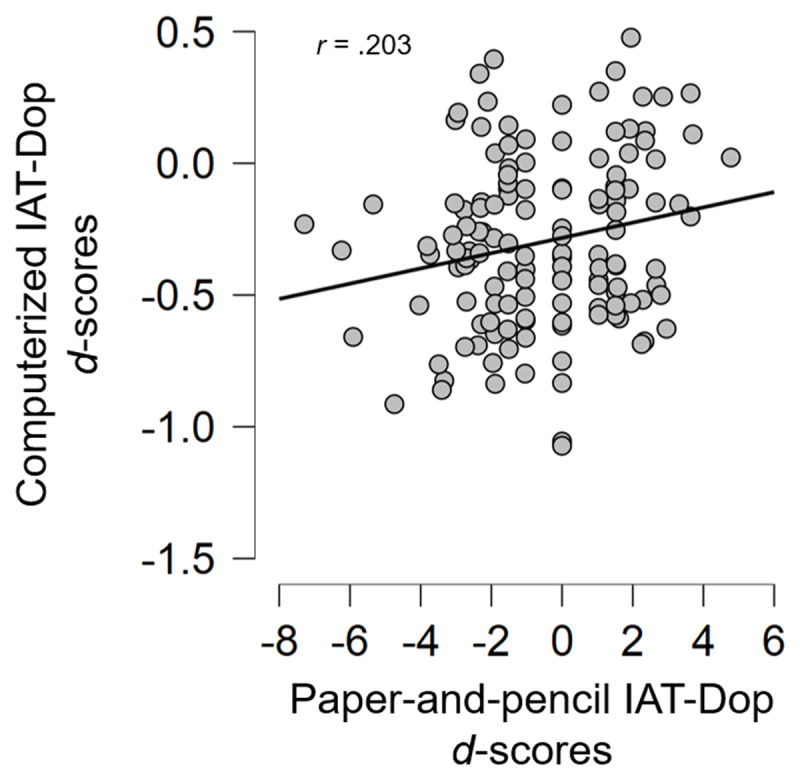
Scatter plot indicating *d*-scores for the two versions of the IAT-Dop: The computerized and the paper-and-pencil.

When comparing the *d*-scores obtained by the total sample with each of the IAT versions, participants obtained an average *d*-score of –0.30 (*SD* = 0.32) with the computerized version of the IAT-Dop and an average *d*-score of –0.49 (*SD* = 2.27) with the p&p version (see Figure 6).

Correlations run between the computerized and p&p versions of the IAT-Dop showed a significant link (*r* = 0.20, *p* = 0.02, with a statistical power of 77%). This result suggested that both the p&p and computerized IAT have the capacity to measure implicit attitudes toward doping.

### Discussion

This study examined the relationship between the p&p IAT-Dop and its equivalent computerized version. Computerized IATs are widely used tools for assessing implicit attitudes and beliefs (see for reviews Greenwald et al., 1998, Lane et al., 2007, Nosek et al., 2007b). The p&p versions have appeared more recently and remain less frequently used. Both versions measure the strength of association between a target and evaluative attributes. Computer-based measures are based on response latencies to categorize the stimuli, while paper-based measures are based on the number of correct answers in a given time. Our results showed a significant correlation between the *d*-scores of the p&p and computerized versions of the IAT-Dop. We observed an effect of sport level on *d*-scores, with higher *d*-scores for international athletes than for national athletes, but only for the computerized version. This effect could be explained by the fact that international athletes were more familiar with doping words and were able to regulate themselves more easily than national athletes with the direct feedback in the computerized version (i.e., a red cross that appeared in the middle of the screen when they committed an error). It can be hypothesized that international athletes learned more quickly from their errors. Furthermore, as for Study 2, we noticed an order effect with higher *d*-scores for participants starting with block A (i.e., Doping + I like) than for participants starting with block B, but only with the p&p version of the IAT-Dop. This result may be explained by the fact that in the p&p version, participants do not benefit from direct feedback on their errors, in contrast to the computerized version (i.e., a red cross appears in the middle of the screen when the participants make an error). Also, we noticed a gender effect on *d*-scores for both IAT versions. Consistent with the literature on doping, females presented lower *d*-scores than males (see for Nicholls et al., 2017, for a review).

Although computerized versions are accurate and suited to collecting latency measures, this p&p version provided undeniable advantages in terms of administration (i.e., time and ease of administration, lower cost, no need for equipment). However, it should be noted that the correlation between the two *d*-scores (i.e., computerized and p&p) is weaker than previously observed by Bardin et al. (2016). Lemm et al. (2008) also presented a moderate effect for the correlation between the two *d*-scores, suggesting that the lack of a strong effect may be due to (a) the substantial error variance associated with the scores and (b) the weakness of paper-format IAT to elicit strong mean effects.

In future work, the p&p IAT-Dop could facilitate the measure of implicit attitudes to enhance our understanding of the psychological mechanisms related to doping. Moreover, this test might be implemented as a new indicator in the evaluation of doping prevention programs.

## General Discussion

The aim of this research was to develop and test a preliminary French version of the paper-and-pencil (p&p) Implicit Association Test (IAT) to measure athletes’ attitudes toward doping: the IAT-Dop (French Paper-and-Pencil Association Test to Measure Athletes’ Implicit Doping Attitude). The development and testing of this scale was carried out in four complementary studies, which followed the testing procedure of Bardin et al. (2016) enriched with several parts of Boateng et al.’s validation recommendations (2018).

In the first study, we developed a preliminary French version of the IAT to measure athletes’ implicit doping attitudes, based on Chan et al.’s IAT proposal (2017). The second study provided preliminary support to the dimensionality and criterion validity of the tool. The third study demonstrated the test-retest reliability of the instrument. The significant correlation between the computerized and p&p versions found in the last study suggests the IAT-Dop is concurrently valid. The IAT-Dop measures athletes’ implicit attitudes, both positive and negative, toward doping in sport (Brand et al., 2014; Chan et al., 2017; Petróczi et al., 2008). It is composed of two valence categories [i.e., positive: *J’aime* (‘I like’) and negative: *Je n’aime pas* (‘I dislike’)] and one target category *Dopage* (‘Doping’), which includes the four stimuli words: (a) *EPO* (‘EPO’), (b) *Transfusion* (‘Transfusion’), (c) *Stéroïdes* (‘Steroids’), and (d) *Corticoïdes* (‘Corticoids’).

In this study, we offer a solid methodological foundation for future studies. Indeed, with the stages of translation and adaptation, we followed a referenced transcultural validation methodology to transculturally validate the IAT. Specifically, we referred to the validation recommendations of Boateng et al. (2018) and the testing of a French p&p SC-IAT-P (Bardin et al., 2016). By presenting mixed methodologies for validating an IAT (i.e., Bardin et al., 2016; Boateng et al., 2018), we thus offer guidelines for the development of IAT translation and adaptation studies in the future.

Beyond our emphasis on the advantages offered by the p&p IAT, our study itself presents some specific strengths. First, validations of p&p IATs remain scarce in the literature, and guidelines are nonexistent. By evaluating the dimensionality and criterion validity (Study 2) and the test-retest reliability (Study 3) of the tool, we added supplemental steps to the transcultural validation process and improved the strength of our results. Also, the sample size of our work was larger than that of similar studies, reinforcing the reliability of the validation. Second, our tool was specially designed for cycling through the semantic research conducted with cycling specialists, but it was then validated with students from a university sports science school and practitioners from various sports. Future studies in a range of sports could therefore use the IAT-Dop in this way or it could be adapted to a specific sport by choosing the doping substances most often used in the concerned sport. Furthermore, even athletes practicing at a departmental or regional level are likely to engage in doping behaviors. Therefore, future research in sport performance, and not only in cycling, might use the IAT-Dop without changing any stimuli or just in adapting the stimuli to the sport specificities. Finally, it’s one of the very first findings in the literature that showed an association between IAT score and doping behavior. This tool will be helpful and is a first step in efforts to better understand the relationships between explicit and implicit attitudes, self-reported doping behavior, and doping behavior.

Nevertheless, some limitations related to this work need to be acknowledged. First, the IAT itself has been widely debated in the literature. Recently, the original authors of the IAT published a state-of-the-art of the best practices for using an IAT (Greenwald et al., 2022) highlighting the difficulty of reaching a consensus. Concerning the computerized IATs, authors agreed that it is necessary to fund solutions to the problem of the inherent noisiness of latency measures. Moreover, despite all the precautions, a participant faking the test would undoubtedly affect its validity and reliability. In our study, the exclusion of every participant presenting error rates > 20% strongly reduced this weakness.

Moreover, because doping is a very sensitive topic, it is important to consider possible explanations for the high error rates obtained. Indeed, although Lemm et al. (2008) obtained an error rate of 30.13% in their test, which serves as a reference for p&p IAT, error rates obtained in our study (i.e., 9.43%) were high compared to other similar studies (Bardin et al., 2016; Lane et al., 2005; Lowery et al., 2001) for example, Bardin et al. (2016) obtained 4.6% in a smaller sample: *n* = 108. A first explanation might come from the fact that doping is a very sensitive topic (i.e., forbidden and not socially acceptable). Interestingly, we noticed that all the responses with error rates >20% (i.e., meeting the exclusion criteria) occurred in block A ‘Doping + I like,’ despite the training phase. Also, all the participants excluded for error rates >20% (in block A), belonged to the ‘non-dopers’ group according to their self-reported behavior. Another explanation could come from the closeness of the target category: while young adults are familiar with tobacco (i.e., Bardin’s target), they are less familiar with doping and all the associated words (e.g., steroids, corticoids). Also, when participants began with block A (doping + I like), they presented higher *d*-scores. This effect of order has frequently been reported in research on IAT (e.g., Clément-Guillotin et al., 2012; Greenwald et al., 2003; Nosek et al., 2005).

Moreover, it was impossible to relate our results of implicit attitudes to objectively measured doping behavior (e.g., measured by blood or urinary tests). Therefore, it was only possible to observe relations between self-reported doping attitudes and behaviors (potentially biased) and implicit attitudes. Even though the sample size in Study 2 was large (*n* = 144), only a relatively small number of participants admitted to being prohibited substance users on the self-report questionnaire (*n* = 29, 20.14%). This small sample size could explain the lack of significance in the difference in implicit attitudes between the two groups (i.e., dopers and non-dopers). This could also be the reason we found, in Study 2, only a tendency in the difference between the implicit attitudes of the two groups (i.e., dopers and non-dopers). Comparing scores of each group to zero, we found no significant difference only for the ‘dopers’ group which can be explained by low positive attitudes to doping (i.e., the floor effect, Ntoumanis et al., 2014). Recent studies on doping have used recruitment strategies to discriminate *a priori* the clubs or structures which present strong doping likelihood and thus limit the floor effect (e.g., Kavussanu et al., 2021, 2022). Comparisons of our results with other papers are difficult because, to our knowledge, no study has ever measured the implicit attitudes towards doping of users who engage in it. The only other study in the literature that includes dopers (Barkoukis et al., 2015) only considered explicit measures without measuring implicit attitudes.

In addition, in our study, the use of the same sample for our Studies 2 and 4, although large and diverse, should stimulate further studies to confirm our results. Also, despite all our precautions, it is possible that some participants included in the ‘dopers’ group, were using prohibited substances (e.g., cannabis) for recreational purposes more than for an ergogenic goal.

The development of the IAT-Dop opens new avenues of research to enrich the doping literature. Methods of psychometric testing based on reaction times—like the IAT-Dop—are necessary to look at implicit mechanisms more closely. Doping studies are particularly at risk of social desirability biases because doping is illicit and not socially acceptable (Blaison et al., 2006; Brand et al., 2014). Moreover, a recent systematic review aimed at identifying the psychological factors related to unintentional doping (Chan et al., 2020). Behavioral, social, and psychological factors were found to be involved in the avoidance of unintentional doping through the variables from the SDT (Ryan & Deci, 2000), the TPB (Ajzen, 1991), and the trait self-control. Therefore, implicit attitudes might predict different facets of doping behaviors. By providing a new French tool to easily measure implicit attitudes toward doping in line with the recent doping application developed by Tang et al. (2022), our study will offer to future research the perspective of complementary results, less distorted by social desirability biases and attempts at faking. Indeed, research on doping has traditionally measured and studied the explicit processes involved in the decision to use prohibited substances.

The identification of both explicit and implicit attitudes toward doping among athletes may be a key factor in doping prevention efforts (Chan et al., 2018). Indeed, according to the literature, especially the dual-process models of health behavior (e.g., Friese et al., 2011; Strack & Deutsch, 2004), taking into account both the traditionally explored explicit mechanisms and the implicit predictors can improve our understanding of behaviors (Sheeran, 2005). Providing a reliable tool capable of measuring implicit attitudes towards doping in sport was therefore a necessity. This p&p version appears to be particularly useful in a sports context (e.g., poolside, athletics field) where using computerized tools is difficult. The p&p versions of IATs will likely lower testing costs because they are easy to run and require little equipment (Vargas et al., 2005), as opposed to computerized versions, which require not only laptops or tablets, but also expensive software. The p&p versions also enable the attitudes of several participants to be measured simultaneously, which saves time for researchers.

From a practical standpoint, this scale will be helpful in efforts to prevent doping as it is not based on the assumption that doping behavior results from a deliberate choice between several alternative solutions. Further, the IAT-Dop is likely to have implications for doping prevention policies and interventions. As it can also provide a reliable assessment indicator of doping prevention strategies, the IAT-Dop should guide all the actors of antidoping efforts in the design of optimal education and prevention programs to prevent doping in sport. The learning of associations between a behavior and affects (positive or negative) present significant results for attitudes (Antoniewicz & Brand, 2016). For example, studies related to health behaviors trained participants with a joystick to approach healthy behaviors and avoid unhealthy ones (e.g., alcohol drinking: Wiers et al., 2011. Their results encourage the development of interventions intended to change the automatic bases of health behaviors. So far, the literature does not provide any application of such strategies to change behaviors in the context of sports performance. This work might contribute to generate further intervention techniques including both traditional explicit techniques (e.g., watching videos, playing games, classroom interventions) and implicit-focused strategies, which have already demonstrated efficacy in health behavior change (e.g., Friese et al., 2011; Wiers et al., 2011).

## Conclusion

The IAT-Dop is a preliminary tested French-language version of a tool for measuring athletes’ implicit attitudes toward doping, with the advantages of simplicity, low cost, and quick administration. We expect it to stimulate future research into the mechanisms related to doping and to serve as a new indicator in the evaluation of prevention programs.
